# Efficacy of behavior modification training combined with electroencephalographic biofeedback therapy for attention deficit hyperactivity disorder in children: a randomized controlled trial

**DOI:** 10.3389/frcha.2023.1235310

**Published:** 2023-11-21

**Authors:** Xiangfen Luo, Ling Zhang, Lei Xia, Xiaoqin Zhou

**Affiliations:** ^1^ Department of Psychiatry, Chaohu Hospital of Anhui Medical University, Hefei, China; ^2^ Department of Psychiatry, The Second Affiliated Hospital of Bengbu Medical College, Bengbu, China

**Keywords:** behavior modification training, EEG biofeedback, therapy, attention deficit hyperactivity disorder, children

## Abstract

**Background and aims:**

Attention deficit hyperactivity disorder (ADHD) is one of the most common psychiatric disorders in children. Multiple treatments are currently available with varying effectiveness, and our aim was to investigate the efficacy of behavior modification training combined with Electroencephalography (EEG) biofeedback treatment on ADHD in children.

**Methods:**

Children with ADHD were randomly divided into a control group (*n *= 42), an EEG biofeedback group (*n *= 30) and a behavior modification training combined with EEG biofeedback group (i.e., a combined intervention group) (*n* = 30) according to the intervention. Swanson, Nolan, and Pelham, Version IV (SNAP-IV) and Conners Parent Symptom Questionnaire (PSQ) were assessed before and after three months of treatment.

**Results:**

We found that in the EEG biofeedback group and the combined intervention group, the scores of all factors except “anxiety” and “psychosomatic disorder” were lower than before treatment, and the difference was statistically significant (*P *< 0.05). After treatment, the scores of the three groups were compared. The scores of “impulsivity-hyperactivity”, “learning problems”, “inattention factor” and “hyperactivity factor” were all lower than before, and the difference was statistically significant (*P *< 0.05). In the post-treatment comprehensive intervention group and the control group, the efficacy was apparent, and the differences in the scores of each factor were statistically significant (*P *< 0.05). In the comparison between the EEG biofeedback group and the control group, except for “anxiety”, “psychosomatic disorder” and “conduct problem” the scores of each factor were statistically significant (*P *< 0.05). For the comparison between the integrated intervention group and the EEG biofeedback group, the scores of all factors before and after treatment were statistically significant (*P *< 0.05), except for “anxiety”, “impulsivity-hyperactivity” and the scores of all the factors before and after treatment were statistically significant (*P *< 0.05), except for “anxiety”, “impulsivity-hyperactivity” and “psychosomatic disorder”.

**Conclusions:**

The comprehensive efficacy of behavior modification training combined with EEG biofeedback therapy on the improvement of symptoms in children with ADHD is positive, and good compliance is worthy of clinical promotion.

**Clinical Trial Registration:**

https://www.chictr.org.cn/indexEN.html, identifier (ChiCTR2300071511).

## 1. Introduction

Attention deficit hyperactivity disorder (ADHD) is one of the common neurodevelopmental disorders. The global prevalence of ADHD in children and adolescents is estimated to be approximately 5% (1–3). Research statistics show that the prevalence of ADHD in children is estimated to be 6.26% in China (4), with a significantly higher prevalence in boys than in girls. ADHD is mostly seen in school-aged children, and its core symptoms include hyperactivity, impulsivity or inattention that are not appropriate for their developmental age. Most of the symptoms persist into adolescence and even adulthood. Since ADHD is often comorbid with other disorders (5–7), such as mood, anxiety, and conduct disorders, it may have a wide and negative impact on the academic, occupational, and social life of patients (8). Therefore, an effective and highly compliant treatment modality is necessary.

The more common and accepted treatment modalities for ADHD are pharmacological and nonpharmacological treatments. Nonpharmacological treatments include EEG biofeedback and behavior modification therapy, which are designed to correct the behavior of children and improve their core symptoms. Clinical studies in the last decade have found (9, 10) that despite the positive efficacy of drug therapy, there may be safety hazards that affect children's development, such as loss of appetite and delayed height development, in the process of drug therapy (1, 11), and due to more parental concerns and resistance to give children long-term medication leading to poorer medication adherence. In addition, there are certain side effects of pharmacological treatments, such as anorexia, sleep disturbances, and headaches (9, 12). Some studies have shown that stimulants in drug therapy could not improve academic performance (13).

In recent years, EEG biofeedback has been widely used in the treatment of ADHD. Studies have found (14, 15) that EEG biofeedback therapy is effective in improving the core symptoms of ADHD. Previous studies also showed (16, 17) that children with ADHD have increased activity of theta waves and decreased activity of beta waves in the prefrontal center, and EEG biofeedback therapy is a method of extracting specific parameters from EEG signals as a reference for brain function training to suppress theta waves and strengthen beta waves (18–20).

With the intensive use of various behavioral therapies, it has been asked whether behavior modification training combined with EEG biofeedback for ADHD is more advantageous than a single treatment. In this study, we focused on the efficacy of behavior modification training combined with EEG biofeedback treatment for children with ADHD from this perspective, aiming to provide a basis for the standardized treatment of ADHD.

## 2. Materials and methods

### 2.1. Study participants

All subjects gave their informed consent for inclusion before they participated in the study. The study was conducted in accordance with the Declaration of Helsinki, and the protocol was approved by the Ethics Committee of Chaohu Hospital of Anhui Medical University (IRB No. 2019-kyxm-012). All children and parents signed an informed consent form. Children with ADHD who attended the outpatient clinic of the Department of Psychiatry of the Second Affiliated Hospital of Bengbu Medical College from July 2020 to Sep 2022 were selected as the study subjects. All children met the diagnostic criteria for ADHD in the Diagnostic and Statistical Manual of Mental Disorders, 5th edition (DSM-5) (21). Excluded were: (i) those who had left compulsory education; (ii) those with combined intellectual disability (IQ <70); (iii) those with severe physical illnesses, chronic infections, and other psychiatric disorders; (iv) children with schizophrenia, mood disorders such as childhood anxiety, depression, autism spectrum disorders, conduct disorders, tic disorders, and other comorbid disorders; (v) children who had taken medication for ADHD.

### 2.2. Research methodology

This study was an exploratory controlled clinical study. According to the preliminary experiment and literature review, the main efficacy index hyperactivity index decreased by about 0.2 ± 0.08 after treatment. We set Power = 0.9 and Alpha = 0.05 (bilateral), the sample size of three groups was 12 cases by PASS calculation. Assuming that the shedding rate of the subjects was 20%, the minimum sample size was 15 cases in each group.

A self-designed general situation questionnaire was used to collect general demographic data of the subjects, the PSQ and SNAP-IV (Parent Version) were evaluated. The groups were numbered according to the order of attendance at enrollment and then divided into a control group (*n *= 42), an EEG biofeedback group (*n *= 32), and an EEG biofeedback combined with behavior modification training group (i.e., combined intervention group) (*n *= 33) using a random number table method. During the study, two children in the EEG biofeedback group were dislodged due to traveling (one child was dislodged after the third biofeedback session and another one was dislodged after the fifth biofeedback session), and 30 cases actually completed; one child in the integrated intervention group was dislodged due to the change of parents’ jobs and the need to move to another city (dislodgment node: after the eighth biofeedback session), and two children were dislodged due to traveling (one of them was dislodged after the sixth biofeedback session, and the other after the eighth biofeedback session), and 30 cases were actually completed; no dislodgment was seen in the control group; 42 cases were actually completed. The treatment plan was formulated after comprehensive assessment of the three groups, in which the control group was not given any intervention; the EEG biofeedback group was given timely biofeedback treatment; and the comprehensive intervention group was given the same course of biofeedback treatment and behavior modification training. After three months of treatment, the scores of impulsivity-hyperactivity, hyperactivity index and SNAP-IV factors of the PSQ scale were assessed again in the three groups.

#### 2.2.1. EEG biofeedback therapy

EEG biofeedback treatment was performed on two groups of children using a multiparameter EEG biofeedback instrument provided by Guangzhou Runjie Medical Equipment Co., Ltd., China. First, children were placed in a sitting position and allowed to quietly, relax in the training room for 3–5 min. Their faces were kept free of perspiration and foreheads were disinfected with alcohol. They were then connected the electrode cap, and given instructional language guidance. The children were encouraged to take the test seriously, and after the baseline test and alpha wave music relaxation, chose the corresponding game training program to suppress theta waves of 4–8 Hz and strengthen beta waves of 12–30 Hz. First, in the relaxation phase, the duration of alpha wave music relaxation was controlled at 3–5 min; second, in the biofeedback treatment phase, 3–5 game animations were selected, the treatment duration of each being controlled at 4–5 min, and each training period controlled at 15–25 min. Treatment was given 2–4 times per week (22, 23).

#### 2.2.2. Behavior modification training

At least two psychiatrists or psychotherapists developed an individualized behavior modification treatment plan according to each child's condition (24–26). The operational procedures and training programs for behavior modification training were as follows: (i) Through interviews with parents to understand the composition of the family, parenting style and the child's personality and behavioral habits, factors affecting the child's inattention were identified, and parents trained, i.e., behavior parent training (BPT) (27–29). The training instructed parents on how to apply the principles of behavior modification in the home environment to improve their child's behavior. Parents had a weekly group session to share their experiences with each other, while the psychotherapist answered questions and solved problems. (ii) One-on-one communication with the affected child to help the child recognize his or her problems and at the same time be able to build confidence and courage to overcome undesirable behavior. (iii) Group attention training: Through different game settings, the child was allowed to understand the rules of the game, follow the rules and perform training activities. At the same time, anti-distraction training was conducted to instruct the child on how to distribute and focus their attention. When positive and appropriate behaviors emerged during training, praise and affirmation were given; when noncompliance with game rules and hyperactivity emerged, activities could be terminated and punished as appropriate, and attention was given to the application of behavior modification methods such as positive reinforcement, negative extinction and temporary isolation. This was especially true when hyperactivity and inattentiveness brought about adverse consequences; 1 to 2 times a week. (iv) Individual attention training: Depending on each child's situation, parents were asked to spend 20–30 min of individual attention training activities with their child at home every day, such as Schulte squares. The training program was regularly adjusted according to the actual achievement of the child.

### 2.3. Assessment tools and efficacy indicators

#### 2.3.1. Self-made general information questionnaire

The homemade general information questionnaire was developed to remove some private information and collect the general information of the subjects, including gender, age, grade, and whether they were only children.

#### 2.3.2. PSQ

The PSQ include the Teacher Questionnaire, Parent Questionnaire and the Parent Teacher Questionnaire (30). The revised parent questionnaire with 48 items was used in this study, including 6 behavioral problem factors: character problems, learning problems, psychosomatic problems, impulsivity-hyperactivity, anxiety, and hyperactivity index. A four-point scoring method from 0 to 3 was used. The factor scores and the hyperactivity index were used to determine which behavioral problems children had and their severity. Each factor score was the average of the scores of the included items. A score of greater than or equal to 1.5 on the hyperactivity index was considered positive for primary screening. The higher the score, the more likely the child was to have ADHD (31). In this study, the parents rated the children separately before and after treatment according to the content of the scale combined with their daily performance, and their main efficacy evaluation indices were the impulsivity-hyperactivity and hyperactivity index. The revised parent questionnaire of PSQ was also applied in many studies (32).

#### 2.3.3. SNAP-IV

SNAP-IV is a commonly used screening tool for children with attention deficit hyperactivity problems and for efficacy assessment (33). The current commonly used version is the SNAP-IV-18, which includes a parent version and a teacher version. In this study, the parent version was used, and the scale included two factors, inattention and hyperactivity/impulsivity, with nine entries per subscale, using a four-point scoring system from 0 to 3. The subjects' total score on each subscale was first calculated, and then the mean value of each scale item (i.e., total subscale score/9) was calculated. The higher the score, the more severe the symptom, and a score of less than 1 was the normal range. If the score was greater than or equal to 1.6, ADHD was identified. If the score was between 1.1 and 1.5, then at least five items must have been scored as 2 (moderate) and/or 3 (severe) to be identified as ADHD (34). The parent version of SNAP-IV was also applied in previous studies (35).

### 2.4. Quality control and safety evaluation

All interventions in this study were performed under the guidance of psychiatrists or psychotherapists. All treatments were carried out in strict accordance with a uniform operational procedure, and the performance and efficacy of the children were recorded in a timely manner, with timely feedback and adjustment of the treatment plan as appropriate. Children who were unable to adhere to behavior modification training or EEG biofeedback treatment were allowed to withdraw from the study. Data entry in this study was performed by double entry to ensure the accuracy of the data entered. The EEG biofeedback treatment used in this study was safe and painless, and the children's compliance was high. Some children experienced itching at the location where the electrode cap was in contact with the forehead, and after adjusting the tightness of the electrode cap, the discomfort disappeared after ten minutes, and no other discomfort reactions were observed. The behavior modification training was mainly a game setting, and the children were very interested and complied well.

### 2.5. Data analysis and statistics

Statistical analyses were performed using SPSS software, version 17.0 (SPSS Inc., Chicago, USA). The variables were expressed as the mean ± standard deviation or frequency. Analysis of dichotomous variables such as the gender distribution of children in the three groups was performed by the chi-square test. For repeated variables, an independent samples *t*-test was used for comparison before and after treatment between two groups, and a paired *t*-test was used for comparison before and after treatment within groups. Analysis of variance (ANOVA) was used for comparisons between multiple groups, and the Least significant difference (LSD) method was used for two-way comparisons between groups. Differences were considered statistically significant at *P *< 0.05.

## 3. Results

### 3.1. General information

A total of 102 children were included in this study, with a mean age of (8.20 ± 1.653) years old: 82 males (80.4%) and 20 females (19.6%); 40 only children (39.2%) and 62 non-only children (60.8%). There were no statistically significant differences in age, sex or distribution of only children among the three groups (*P *> 0.05). There were no significant differences in the scores of the factors in the PSQ and SNAP-IV-18 between the three groups before treatment, as shown in Table 1.

**Table 1:** Comparison of general data distribution and baseline PSQ and SNAP-IV scales among the three groups of children.

Variables		Control group (*n *= 42)	EEG Biofeedback Group (*n *= 30)	Integrated intervention group (*n *= 30)	X^2^/*F*/*t*	*P* *
Age (year)		8.14 ± 1.72	8.53 ± 1.55	7.93 ± 1.66	0.437^a^	1.025
Gender	Male	34	23	25	1.025^b^	0.362
	Female	8	7	5		
Only child	Yes	13	13	14	2.315^b^	0.347
	No	29	17	16		
PSQ						
Hyperactivity index		1.87 ± 0.35	1.97 ± 0.38	1.88 ± 0.38	0.739^c^	0.480
Anxiety		0.07 ± 0.15	0.15 ± 0.39	0.17 ± 0.26	1.509^c^	0.226
Impulsive-hyperactivity		1.42 ± 0.42	1.40 ± 0.57	1.59 ± 0.36	1.665^c^	0.194
Psychosomatic disorders		0.17 ± 0.40	0.22 ± 0.46	0.02 ± 0.06	0.965^c^	0.385
Learning issues		2.26 ± 0.44	2.34 ± 0.52	2.29 ± 0.48	0.245^c^	0.783
Character issues		1.25 ± 0.47	1.36 ± 0.45	1.24 ± 0.54	0.503^c^	0.606
SNAP-IV						
Inattention factor		2.04 ± 0.36	2.04 ± 0.50	1.76 ± 0.47	0.675^c^	0.512
Hyperactivity factor		1.57 ± 0.57	2.14 ± 0.40	1.54 ± 0.74	1.207^c^	0.303

Data were expressed as the mean ± standard deviation or *n*; The PSQ and SNAP-IV were used for baseline assessment in children with ADHD.

^a^ Statistical values were expressed as *t*-values.

^b^ x^2^.

^c^ ANOVA.

* *P* > 0.05.

### 3.2. Comparison of PSQ and SNAP-IV-18 scales before and after treatment in the EEG biofeedback and comprehensive intervention groups

After three months of intervention, no significant improvement was seen in the control group. A comparison between the EEG biofeedback group and the combined intervention group showed: (i) For PSQ, the scores of impulsivity-hyperactivity, hyperactivity index, learning problems and conduct problems were significantly lower than those before treatment, and the differences were statistically significant [e.g., hyperactivity index in the EEG biofeedback group (1.97 ± 0.38) vs. (1.45 ± 0.37), impulsivity-hyperactivity (1.40 ± 0.57) vs. (1.09 ± 0.56), hyperactivity index in the combined intervention group (1.88 ± 0.38) vs. (0.95 ± 0.42), impulsivity-hyperactivity (1.59 ± 0.36) vs. (0.86 ± 0.44), *P* < 0.05]; (ii) for SNAP-IV-18, the scores of inattention factor and hyperactivity factor in both groups were significantly lower than before The differences were statistically significant [e.g., inattention factor (2.04 ± 0.50) vs. (1.44 ± 0.46) in the EEG biofeedback group, inattention factor (1.76 ± 0.47) vs. (1.25 ± 0.48) and hyperactivity factor (1.54 ± 0.74) vs. (0.64 ± 0.53) in the combined intervention group, both *P *< 0.05], as shown in Table 2.

**Table 2:** Comparison of PSQ and SNAP-IV-18 factor scores before and after treatment in the EEG biofeedback and comprehensive intervention groups.

Variables	EEG Biofeedback Group (*n *= 30)		*t* **	*P*	Integrated intervention group (*n *= 30)		*t* **	*P*
	Before treatment	After treatment			Before treatment	After treatment		
PSQ								
Hyperactivity index	1.97 ± 0.38	1.45 ± 0.37	10.771	<0.001*	1.88 ± 0.38	0.95 ± 0.42	24.217	<0.001*
Anxiety	0.15 ± 0.39	0.17 ± 0.29	0.465	0.645	0.17 ± 0.26	0.07 ± 0.11	2.11	0.043*
Impulsive-hyperactivity	1.40 ± 0.57	1.09 ± 0.56	5.656	<0.001*	1.59 ± 0.36	0.86 ± 0.44	17.012	<0.001*
Psychosomatic disorders	0.22 ± 0.46	0.15 ± 0.32	1.439	0.161	0.02 ± 0.06	0.01 ± 0.04	1.439	0.161
Learning issues	2.34 ± 0.52	1.57 ± 0.46	10.441	<0.001*	2.29 ± 0.48	1.28 ± 0.51	16.046	<0.001*
Character issues	1.36 ± 0.45	1.20 ± 0.45	5.326	<0.001*	1.24 ± 0.54	0.67 ± 0.49	11.930	<0.001*
SNAP-IV								
Inattention factor	2.04 ± 0.50	1.44 ± 0.46	10.77	<0.001*	1.76 ± 0.47	1.25 ± 0.48	7.02	<0.001*
Hyperactivity factor	2.14 ± 0.40	0.87 ± 0.56	20.15	<0.001*	1.54 ± 0.74	0.64 ± 0.53	11.67	<0.001*

The PSQ and SNAP-IV scores for each factor were expressed as the mean ± standard deviation.

* *P* < 0.05.

** Statistical analysis was expressed as *t*-value.

### 3.3. Comparison between the three groups before and after treatment

After three months of treatment, we found that (i) compared among the three groups, the scores of “hyperactivity index”, “impulsivity-hyperactivity”, “learning problems”, “inattention factor” and “hyperactivity factor” were lower than those before treatment, and the differences were statistically significant (*P *< 0.05). (ii) Comparing the combined intervention group and the control group, the efficacy was significant and the differences in the scores of the factors were statistically significant [e.g., impulsivity-hyperactivity (0.86 ± 0.44) vs. (1.41 ± 0.41), conduct problems (0.67 ± 0.49) vs. (1.25 ± 0.46), (all *P *< 0.05)]. (iii) Comparing the EEG biofeedback group and the control group, the scores of all the factors before and after treatment were statistically significant except for “anxiety”, “psychosomatic disorders” and “conduct problems” [e.g., hyperactivity factor (0.87 ± 0.56) vs. (1.59 ± 0.56), learning problems (1.57 ± 0.46) vs. (2.24 ± 0.42), all *P *< 0.05]. (iv) The integrated intervention group and the EEG biofeedback group, the scores of all factors before and after treatment were statistically significant, except for “anxiety”, “impulsivity-hyperactivity”, and “psychosomatic disorders”. (e.g., hyperactivity index (0.95 ± 0.42) vs. (1.45 ± 0.37), inattention factor (1.25 ± 0.48) vs. (1.44 ± 0.46), *P *< 0.05). As shown in Table 3.

**Table 3:** Comparison of the scores of each factor between the three groups after treatment.

Variables	Control group (*n *= 42)	EEG Biofeedback Group (*n *= 30)	Integrated intervention group (*n *= 30)	*F*	*P*
PSQ					
Hyperactivity index	1.85 ± 0.35	1.45 ± 0.37^a^	0.95 ± 0.42^b,c^	50.145	<0.001*
Anxiety	0.17 ± 0.23	0.17 ± 0.29	0.07 ± 0.11^b^	2.265	0.109
Impulsive-hyperactivity	1.41 ± 0.41	1.09 ± 0.56^a^	0.86 ± 0.44^b^	12.606	<0.001*
Psychosomatic disorders	0.14 ± 0.31	0.15 ± 0.32	0.01 ± 0.04^b^	3.043	0.052
Learning issues	2.24 ± 0.42	1.57 ± 0.46^a^	1.28 ± 0.51^b,c^	41.433	<0.001*
Character issues	1.25 ± 0.46	1.20 ± 0.45	0.67 ± 0.49^b,c^	15.378	<0.001*
SNAP-IV					
Inattention factor	2.02 ± 0.32	1.44 ± 0.46^a^	1.25 ± 0.48^b,c^	60.054	<0.001*
Hyperactivity factor	1.59 ± 0.56	0.87 ± 0.56^a^	0.64 ± 0.53^b,c^	28.169	<0.001*

Data were expressed as the mean ± standard deviation.

^a^ The EEG biofeedback group compared with the control group, *P *< 0.05.

^b^ The Integrated intervention group compared with the control group, *P *< 0.05.

^c^ The Integrated intervention group compared with the EEG biofeedback group, *P *< 0.05.

* Comparison between the three groups, *P *< 0.05.

## 4. Discussion

In this study, we found that behavior modification training combined with EEG biofeedback therapy was effective in the treatment of children with ADHD. The ADHD is a common neurodevelopmental disorder in childhood, which brings a heavy burden to patients, their families and society. The side effects of medication prevent children with ADHD and their families from taking medication (10, 36). Therefore, parents prefer to choose non-pharmacological treatment with high safety and few side effects.

In this study, the comparison between the EEG biofeedback group and the control group before and after treatment revealed that the factors of “impulsivity-hyperactivity”, “inattention factor”, and “learning issues” improved significantly. This indicated that biofeedback treatment can improve inattention and hyperactivity symptoms, which has been confirmed in many studies (37, 38). However, there was no significant improvement in “anxiety”, “psychosomatic disorders” and “conduct problems”, which may be related to co-morbidities. It has been shown (39) that the core symptoms of ADHD are associated with a large number of psychiatric disorders such as behavioral disorders, learning disorders, anxiety disorders, and sleep disorders. Due to the high rate of co-morbidity, it may complicate not only the clinical presentation of ADHD but also the selection of the most appropriate treatment strategy (40, 41). Therefore, other comprehensive treatment options are needed to compensate, such as behavior modification training and family therapy. Whereas EEG biofeedback training is also a process of operant conditioning, children may suffer from decreased interest and burnout due to repetitive training, which may affect the efficacy. And behavior modification training seems to make up for such shortcomings.

Previous studies (42–44) have shown that behavior modification training can significantly improve hyperactive or inattentive behaviors in children with ADHD. In this study, the treatment effect of the integrated intervention group was significantly better than that of the EEG biofeedback treatment group, especially in the areas of “hyperactivity index”, “inattention”, “learning problems” and “behavioral problems”. It indicates that the children's learning problems and behavioral symptoms were also synergistically improved, which was consistent with the study of Roy S et al. (45). The comparison between the EEG biofeedback group and the comprehensive intervention group revealed that the single EEG biofeedback training may lead to decreased interest, visual and psychological fatigue, and even passive resistance in some of the children due to repetitive training, which in turn may affect the efficacy. The integrated intervention group used behavioral modification training combined with EEG biofeedback treatment, and a series of game training was set up in the behavioral modification training, which resulted in a high sense of interest and participation of the children, greatly reducing the fatigue of the children, improving the children's adherence to the treatment, and enhancing the confidence of the children and their parents in adhering to the treatment.

In addition, the target of behavior modification in our study is not limited to children, but also parents, i.e., behavioral parent training (BPT). As we know, the etiology of ADHD is not only genetic, but also socio-family psychological factors, especially poor parental character and parenting style of the family. And the limitations of children's personality traits, behavior modification must require parental guidance and supervision. And BPT is used clinically as a proven, evidence-based treatment for pediatric ADHD (46, 47). It is particularly effective for children with ADHD who have disruptive behaviors (48, 49). In the study, parents gave weekly feedback on their children, and most parents reported that parent training helped children with personality problems, which can be quantified in future studies. For adolescents with ADHD, BPT has been categorized as a potentially effective treatment. This is consistent with the findings of Sibley et al. (2016) (50, 51).

Overall, in this study, the advantages of both EEG biofeedback therapy and behavior modification training include high safety, small adverse reactions, and long-term application. The combined use of the two not only makes the treatment more diversified, but also effectively improves the treatment compliance of the children. Through the training, the bad behavioral habits subsided, the good behavioral habits were strengthened, the core symptoms improved significantly, and the learning efficiency was improved.

Nevertheless, the study has some limitations. Firstly, the follow-up time is relatively short. Studies by some scholars in China have shown (52) that after one year of behavioral intervention, anxiety disorders and psychosomatic disorders associated with ADHD can be further improved. Subsequent follow-up can be continued by extending the follow-up time, etc., which may provide a stronger indication of clinical efficacy. Second, in this study, the validity of clinical symptoms was assessed mainly by parent questionnaires. Due to the epidemic and the summer vacation in China, it was not practical for teachers to participate in the assessment. Of course, if the parent questionnaire and the teacher questionnaire can be assessed at the same time, then it can be better to provide feedback on the effectiveness of the treatment. More systematic and objective clinical assessment tools will be added to the study in the future. Meanwhile, the intervention targets of the behavior modification treatment in this study were mainly parents and children. Subsequent trial design may consider adding teacher training for school intervention, which may be more conducive to the improvement of ADHD clinical symptoms. Finally, there are many comorbidities of ADHD, and the selection of subjects in this study excluded the comorbidity sample, the results of this study may not be generalized to children with ADHD with comorbidities. In the future, the sample size should be further expanded to extend the treatment methods to other ADHD children with comorbidities.

In conclusion, the comprehensive efficacy of behavior modification training combined with EEG biofeedback therapy on the improvement of symptoms in children with ADHD was positive, and good compliance is worthy of clinical promotion.

## Data Availability

The original contributions presented in the study are included in the article/Supplementary Material, further inquiries can be directed to the corresponding author.
